# Identifying ultrasensitive HGF dose-response functions in a 3D mammalian system for synthetic morphogenesis

**DOI:** 10.1038/srep39178

**Published:** 2016-12-16

**Authors:** Vivek Raj Senthivel, Marc Sturrock, Gabriel Piedrafita, Mark Isalan

**Affiliations:** 1 Department of Life Sciences, Imperial College London, London SW7 2AZ, United Kingdom; 2 EMBL/CRG Systems Biology Research Unit, Centre for Genomic Regulation (CRG), The Barcelona Institute of Science and Technology, Dr. Aiguader 88, Barcelona 08003, Spain; 3 Universitat Pompeu Fabra (UPF), Barcelona, Spain; 4 Department of Biochemistry and Cambridge Systems Biology Centre, University of Cambridge, Cambridge CB2 1GA, UK

## Abstract

Nonlinear responses to signals are widespread natural phenomena that affect various cellular processes. Nonlinearity can be a desirable characteristic for engineering living organisms because it can lead to more switch-like responses, similar to those underlying the wiring in electronics. Steeper functions are described as *ultrasensitive*, and can be applied in synthetic biology by using various techniques including receptor decoys, multiple co-operative binding sites, and sequential positive feedbacks. Here, we explore the inherent non-linearity of a biological signaling system to identify functions that can potentially be exploited using cell genome engineering. For this, we performed genome-wide transcription profiling to identify genes with ultrasensitive response functions to Hepatocyte Growth Factor (HGF). We identified 3,527 genes that react to increasing concentrations of HGF, in Madin-Darby canine kidney (MDCK) cells, grown as cysts in 3D collagen cell culture. By fitting a generic Hill function to the dose-responses of these genes we obtained a measure of the ultrasensitivity of HGF-responsive genes, identifying a subset with higher apparent Hill coefficients (e.g. MMP1, TIMP1, SNORD75, SNORD86 and ERRFI1). The regulatory regions of these genes are potential candidates for future engineering of synthetic mammalian gene circuits requiring nonlinear responses to HGF signalling.

Cells constantly gather information from their surroundings and process it to optimise growth, metabolism and fitness. In doing so, genes respond to various external stimuli in dose-dependent manners. Sensitivity, which is defined as the minimum change in input required for maximal change in output, is thus an important parameter to measure the response of such systems to external stimuli^1^. If small changes in input lead to great changes in output, a system is said to be more sensitive, and cells exploit functions of different sensitivity to optimise gene expression and survival.

Sigmoidal dose-responses are very common in biology and as they become steeper, more nonlinear, and switch-like, they are said to be *ultrasensitive*^1^,^2^. The key features of an ultrasensitive response function are thresholding, saturation and a steep response to a small range of input above the threshold (Fig. 1a). While thresholding helps to filter out input noise, saturation defines an upper limit for the output. Mathematical modelling has guided our understanding of the biological implications of ultrasensitive responses, and the main features are captured well by the Hill function^3^,^4^,^5^. Within this function, the Hill coefficient is particularly useful as a parameter that measures the strength of ultrasensitivity^6^,^7^,^8^: a Hill coefficient greater than one indicates that the system has ultrasensitivity^1^ (Fig. 1b).

In terms of biology, ultrasensitivity is essential in situations where all-or-none types of information transfer are required^1^. In fact, ultrasensitivity has been shown to be important in many aspects of cellular signalling^2^,^9^. For example, sensitivity amplification can ensure the faithful transfer of information in a signalling cascade^10^, both in the presence and absence of intrinsic noise^11^,^12^,^13^. In addition, ultrasensitivity can increase the robustness of bistable responses^14^,^15^,^16^ and can stabilise biological oscillations^17^,^18^,^19^,^20^,^21^,^22^,^23^,^24^,^25^,^26^. Finally, ultrasensitivity has been shown to play a major role in converting spatial gradients into sharp boundaries in morphogenesis and developmental patterning^27^,^28^,^29^,^30^,^31^,^32^,^33^. In the latter context, our previous work showed that steeper dose-response functions better supported Turing pattern formation in a theoretical developmental model^28^.

Because ultrasensitivity can be a requirement for the correct functioning of genetic circuits with particular dynamical behaviours or patterning, engineering ultrasensitivity has become an important design objective within synthetic biology^5^,^12^,^34^,^35^. There are at least four major mechanisms by which cells can achieve an ultrasensitive response and these can be categorised as zero order ultrasensitivity, multistep mechanisms, stoichiometric inhibitors and positive feedback loops (described in detail in refs 36 and 37). Consequently, in many synthetic biology studies ultrasensitivity has been introduced artificially into cell signalling networks using these mechanisms. For instance, ultrasensitivity can be induced by increasing the cascade length in a signalling network^12^. Alternatively, by employing a mixture of receptor decoys, small amounts of high-affinity sinks for the signalling molecules can create thresholds, while low affinity decoys force steeper response functions^38^. Similarly, the sequestration of a transcription factor on a dominant negative inhibitor is able to convert a graded transcriptional response to an ultrasensitive response^39^. Other approaches include introducing multiple cooperative binding sites on an enzyme so that a Michaelian response can be converted to an ultrasensitive response^40^. Finally, by using two positive feedback loops, a signalling system with a hyperbolic response curve can also be converted into switch–like response^41^,^42^.

In this study, we aim to identify natural gene regulatory functions that potentially respond in an ultrasensitive manner to input signals, such as those from a growth factor. This information can in theory be used to develop genome engineering tools that can exploit these promoter functions, not as modular interchangeable parts, but in the context of modifying the endogenous chromosomal gene loci, for example by exchanging open reading frames (ORFs). This would prevent context-dependent effects on the function of a promoter part (e.g. ultrasensitivity) via mechanisms such as the position of the promoter-ORF fragment on the genome, epigenetic effects, etc. In this work, we limit our scope to identifying suitable promoter regions and chromosomal integration loci for integrating protein-coding sequences that need ultrasensitive responses. Testing such systems will require further work and will likely require optimization on a case-by-case basis, to engineer functional synthetic networks.

Previously, we developed a toolkit of mammalian cells and growth factors for engineering signalling networks, based on Madin-Darby canine kidney (MDCK) cells. The toolkit is designed as a scaffold for engineering synthetic morphogenesis and pattern formation, based on reaction-diffusion^28^,^43^. The components include Hepatocyte Growth Factor (HGF) as an activator of the c-Met receptor^44^, and a truncated form of HGF called NK4^45^, which acts as an inhibitor on the same receptor. The system lends itself to generating patterns, analogous to those in developmental processes. By linking the effectors to suitable HGF-responsive promoters, the resulting gene circuits were in theory capable of supporting Turing pattern (TP) formation^28^. However, according to the mathematical model, TPs were formed over much larger parameter spaces when the HGF promoter functions had higher apparent Hill coefficients, n (minimum n > 1.33^28^). Therefore, identifying genes with ultrasensitive responses could provide us with more suitable candidate promoter-enhancers for expressing HGF and thus engineering interesting spatiotemporal gene expression patterns.

Following on from this previous work, here we aim to identify ultrasensitive HGF dose-response gene expression functions by genome-wide transcriptome profiling in MDCK cells. We measure the expression levels of 24,580 genes using high throughput RNA sequencing (RNA-Seq), in samples exposed to increasing doses of HGF. After identifying differentially-expressed genes by applying a process of filtering, we obtained a set of responsive genes with monotonous increases in gene expression for increasing doses of HGF. By fitting a generic Hill function to the filtered set of genes (showing sigmoidal dose response and low basal expression) we measure the ultrasensitivity of these genes. Ultimately, this dataset provides a set of HGF gene regulatory functions in MDCK cells grown in 3D collagen cell culture. As well as providing potential promoter-enhancer candidates for gene circuit engineering, the dataset also provides insights into which HGF-responsive genes are more switch-like in deciphering signalling inputs. This has implications for cancer biology, where HGF signalling can play an important role^46^.

## Results

### RNA sequencing to identify HGF-responsive genes in MDCK cells grown in 3D cell culture

#### MDCK and HGF

HGF is a growth factor involved in epithelial cell dedifferentiation, carcinogenesis and metastasis^47^. Because of this, the transcriptome profile that it induces has been previously explored in canine MDCK cells grown in both 2D cell culture and 3D cell culture^48^,^49^,^50^,^51^. These transcriptome studies identified HGF-responsive genes, such as matrix metalloproteinase 1 (MMP1), but used single high doses of HGF (e.g. 100 ng/ml for 24 h^51^) and were not designed to reveal the dose-response characteristics of these genes.

We chose to profile the response of MDCK cells to increasing doses of HGF in 3D culture because any dose-response functions we obtained could thus be applied to our previous synthetic biology work under these conditions. MDCK cells form tissue-like polarised epithelial structures when grown in 3D, in collagen or matrigel, typically forming spherical cysts enclosing fluid-filled lumens^52^. As mentioned above, these conditions are the basis for an engineering scaffold that we previously developed for studying mammalian cell patterning^43^.

### RNA-seq mapping and FPKM values

To test the HGF dose-responses of genes in 3D culture, MDCK cysts were grown in collagen extracellular matrix, for a period of 7 days, and were then treated with variable HGF concentrations. Six different conditions, with two replicates each, corresponding to increasing HGF doses (range 0 to 16.7 ng/ml), were sampled and subjected to RNA-seq analysis (see Methods).

**Figure 1:**
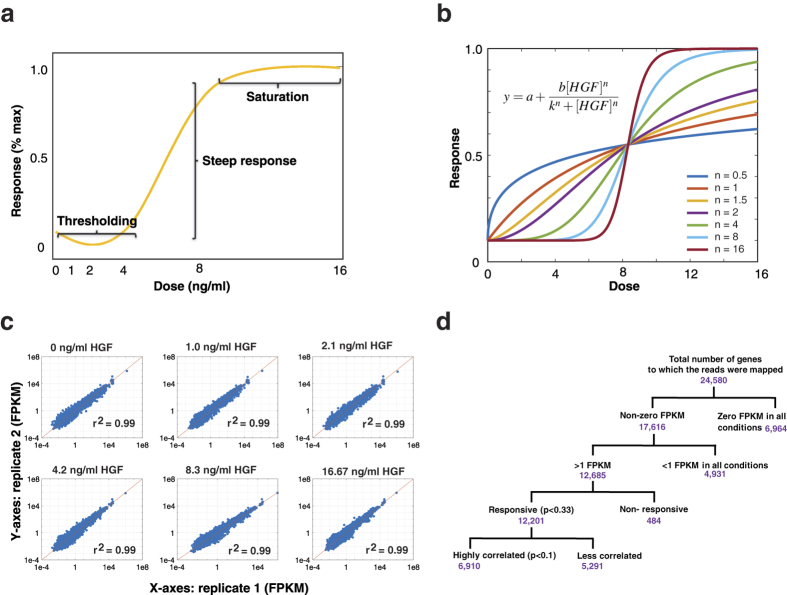
Strategy for identifying ultrasensitive HGF-responsive genes. (**a**) Schematic of an ultrasensitive dose-response function showing features of the curve corresponding to thresholding, steep response and saturation. (**b**) Simulation of dose-response function showing the effect of varying Hill coefficient on the sensitivity of the response. The other parameter values of the Hill function used for the simulation were, *a* = 0.1, *b* = 0.9, *k* = 8.33. (**c**) Log-log scatterplot of FPKM data of all Canine gene models for the two biological replicates (blue solid dots). The red line shows the line of regression through the data and the correlation coefficient is shown as inset (r^2^). (**d**) Number of genes obtained (in purple) at each stage of the filtering process applied to the set of annotated genes, to obtain responsive genes with a very high correlation between replicates.

Total RNA was isolated 12 hours after induction, for two biological replicates, and raw reads of 50-bases were obtained from RNA sequencing, as described in the Methods section. The total number of reads per sample was around 40 million and was mapped to 24,580 annotated genes on the canine genome, from the CanFam3.1 database of ENSEMBL. On average, 71% of the reads (31.5 million) mapped to these annotated genes (after they had passed a quality control step through FastQC: http://www.bioinformatics.babraham.ac.uk/projects/fastqc/). Generally, 20–30 million reads are sufficient for differential gene expression studies with transcriptome data^53^, and the results above are within this range. We then used Cufflinks software^54^ to calculate the relative abundances of the reads in terms of Fragments Per Kilobase of transcript per Million mapped reads (FPKM) values. Out of the 24,580 annotated genes, 17,616 (71.6%) had non-zero FPKM in at least one of the six conditions (Supplementary File 1, Sheet 1). This gives an indication of the large number of genes that can be expressed by MDCK cells. The two biological replicates were correlated by linear regression (Fig. 1c; R^2^ = 0.99) and indeed this correlation was supported by a hierarchical clustering analysis across conditions (Fig. S1).

### FPKM density distribution

The frequency of FPKM values across all expressed genes showed a characteristic bimodal distribution (Fig. S2). The bimodality corresponds to genes which are above or below 1 FPKM, which in turn corresponds to whether genes have more or less than ~1 mRNA per cell on average^55^,^56^. We found that there were fewer genes in the low-expressing group, and more high-expressing genes across all conditions, for both of the replicates. Again, this indicates that the majority of genes are active in MDCK cells grown as cysts.

### Identifying HGF-responsive genes

To find responsive genes out of the pool of 17,616 non-zero FPKM mapped genes, we followed a filtering workflow (Fig. 1d). As shown in the filter tree, we filtered out the low-expressing genes (low expressing in all 6 conditions) using a cut-off value of 1 FPKM. The threshold value of 1 FPKM was selected because it separates the low expressing gene pool from the high expressing one (Fig. S2), and because previous studies give an estimate of approximately 1 mRNA per cell on average for a value of 1 FPKM^55^,^56^. Broadly, the cut-off ensured that we only chose genes with clearly detectable expression in at least one condition; these are more useful for downstream synthetic biology applications. Based on this criterion, we selected 12,685 active genes (Supplementary File 1, Sheet 2). These correspond to 52% of all annotated genes and 72% of the genes with non-zero FPKM values. Therefore, over half of annotated genes were found to be active above 1 FPKM in at least some conditions in MDCK cells grown as cysts.

Next, HGF-induced differentially-expressed genes (DEGs) were identified using a non-parametric statistical test (Kruskal-Wallis)^57^, comparing the five HGF conditions to the one with no induction (0 ng/ml HGF). CuffDiff is a popular method for finding differentially expressed genes and is very effective when measured between two conditions^58^, but we were interested in capturing monotonous increases over a series of conditions. Hence, we preferred using a general non-parametric test with relatively permissive thresholds. Since the main purpose was to use the RNASeq data for visualizing global patterns of response to HGF, mainly as a pre-validation tool, it was appropriate to maximize the identification of all differentially expressed genes (at the expense of a higher false discovery rate). In this sense, CuffDiff is more conservative and underestimates differential expression^59^. The Kruskal-Wallis cut-off of p < 0.33 was the best compromise to avoid discarding slightly differentially expressed genes with high correlation patterns (high consistency) between the two replicates (Pareto front). It is noteworthy to say that more refined non-parametric models inspired in Kruskal-Wallis test have been recently applied with good performance to analyze differential expression from disperse RNA-Seq data^60^,^61^ and in other applications such as multiple-biomarker classification^62^. From the 12,685 high-expressing genes (>1 FPKM in at least one of the five HGF induced conditions), 12,201 genes showed a differential expression across conditions (Fig. S3). These 12,201 responsive genes (Supplementary File 1, Sheet 3) correspond to 70% of the mapped genes with non-zero FPKM and 49% of the total 24,580 genes tested. This shows that a fairly large set of genes is induced by HGF. These gene functions can be broken down into a set of broad cell processes by gene ontology analysis.

The most enriched gene ontology groups based on biological processes in the set of 12,201 responsive genes, belonged to metabolic process, cellular process, biological regulation and localization (73% of the total biological processes) (Table 1). Gene ontology (GO) terms were over-represented and highly enriched for processes such as DNA replication, tRNA metabolic process, and mitotic nuclear division (Supplementary File 3, Sheet 1). A previous study reported that only 3,878 differentially expressed genes (19% of the total genes tested on a canine Affymetrix array) showed at least a 2-fold difference compared to non-induced MDCK cysts^51^. The difference between our results and this much lower number of DEGs could be for several reasons. First, the experimental set up was different in the previous study^51^: the cysts were grown for over 2 weeks and exposed to 100 ng/ml of HGF for 24 hours. By contrast, we used much younger cysts (7 days old), which may be more transcriptionally active than older ones. Second, we tested expression after 12 hours and it is possible that the differential expressions become fewer by 24 hours. Third, we consider genes to be showing significant differential expression even if this is only in one of the five HGF induced conditions; this is a much broader range of conditions compared to the previous study with only one HGF-induced condition. Finally, RNA-seq is more sensitive than microarray methods and so our results would be expected to reveal more potential HGF-responsive gene candidates than were previously measured.

### Principal component analysis of the HGF-triggered response

To assess the overall similarity between the six HGF-concentration conditions with respect to differential gene expression, and to find groups of genes with similar attributes, we performed principal component analysis (PCA) for the set of responsive genes^63^. With PCA we could collapse the six-dimensional data (corresponding to the different HGF induced conditions) to a reduced set of variables that could explain most of the variance in the data. These principal components can be visualised on 2-D or 3-D plots with the principal components as the axes, depending on how many axes are needed to explain most of the data.

**Figure 2:**
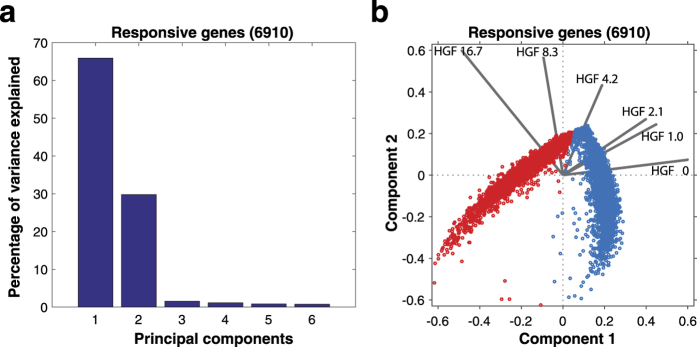
Principal component analysis of responsive genes (6,910). (**a**) Percentage of variance explained by various components. The first component could explain 65% of variance in the data, whereas the second component could explain about 30% of variance in the data. (**b**) Biplot showing component scores (red dots, blue dots) of all the genes in a 2D plane of component 1 and component 2, and vector amplitudes (black lines) of the 6 conditions with respect to the first two principal components.

PCA showed that the first two principal components could explain 68% and 30% of the variance in the data, respectively (Fig. 2a) and that the other components contributed relatively little explanatory power. Hence, we plotted the data on a 2 dimensional biplot (Fig. 2b). The biplot displays two sets of information: one in which the expression of all genes (red and blue dots) are displayed as component scores with respect to the first two principal components; the other set has the contribution of all six conditions to the two principal components as shown as vectors (black lines). In this way, the biplot allows visualization of the magnitude of contribution of the six conditions to the principal components and how the expression of each gene is represented in terms of those components. As a result, we found that the six HGF conditions showed different patterns of gene expression compared to one another, as shown by the vectors in the biplot which are pointing in different directions (Fig. 2b). There is a gradual anticlockwise shift from 0 ng/ml HGF, pointing towards east, to 16.7 ng/ml HGF pointing towards north-west. Based on the length and direction of the vectors, we can infer that 0 ng/ml HGF and 16.7 ng/ml HGF are the two conditions that contribute the most to PC1 and PC2, respectively. This shows that the gene expression patterns of the two conditions are very different, with a gradual change corresponding to increasing doses of HGF. Furthermore, we found two clusters in the PCA plot representing variability due to 0 ng/ml HGF and 16.7 ng/ml HGF (Fig. 2b), for all the mapped genes. These two clusters represent the upregulated genes (red dots, in the direction of 16.7 ng/ml HGF) and the downregulated genes (blue dots, in the direction of 0 ng/ml HGF). The two clusters were very distinct when compared to a PCA analysis done for all the genes (24,580) (Fig. S4), suggesting that the filtering process used to obtain responsive genes was very efficient.

### Hierarchical clustering and functional annotation of HGF-responsive genes

Having found groups of upregulated and downregulated genes using PCA, we next characterised the dose-response functions of the set of responsive genes in more detail, by grouping them according to similarity of behaviour, using hierarchical clustering. This aimed at identifying a range of qualitatively different HGF-induced dose-response functions. Ultimately, these functions could be used to drive different gene expression dynamics so as to engineer synthetic genetic circuits^43^.

**Figure 3:**
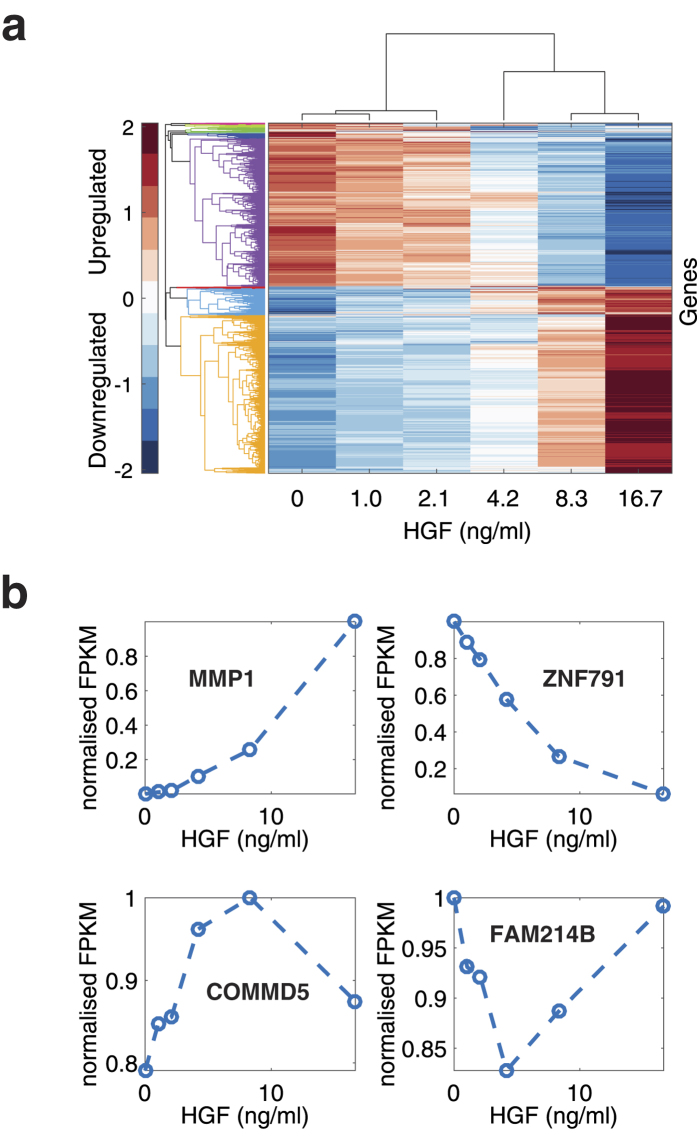
Hierarchical clustering of HGF responsive genes. (**a**) Hierarchical clustering of mapped, high-expressing, correlated, HGF-responsive genes (6,910), using a squared Euclidean distance metric. Responsive genes are first normalised relative to their maximum FPKM value across the different experimental conditions and the average is taken of the two biological replicates. Each row corresponds to a different gene (see Supplementary File 2 for names and raw data) and each column corresponds to an experimental condition. The rows and columns are displayed in the order given by the clustering output trees in the two dimensions. The colour display encodes the logarithm of the normalised expression changes, where red is upregulation, blue is downregulation, and white represents no change. Branches (left), whose linkage distance is less than a threshold of 4, are given the same colour. There are two major clusters, one containing genes displaying monotonous increase with increasing HGF concentration (3,527) and the other with monotonous decrease (2,511). (**b**) Sample response patterns for genes with four different types of responses. MMP1 (monotonous increase), ZNF791 (monotonous decrease), COMMD5 (local maximum; stripe), FAM214B (local minimum; anti-stripe).

To enable hierarchical clustering, we first filtered for genes by correlation between replicates, resulting in 6,910 out of the 12,201 responsive genes, (Supplementary File 1, Sheet 4; a cutoff of *p* < 0.1 was used to exclude highly-variable genes while preserving truly responsive genes). The FPKM values of each gene was normalised to the maximum FPKM value across conditions to prevent the magnitude of FPKM values dominating the clustering, rather than the shape of the dose response (Supplementary File 2). This dataset was used as a basis for the hierarchical clustering (Fig. 3a).

The clustering revealed several classes of dose-response function, including: i) monotonous increase (activation); (ii) monotonous decrease (repression), (iii) local maximum (stripe); local minimum (antistripe) (Fig. 3b). Out of the 6,910 genes, over 51% (3,527) showed a monotonous increase; over 36% (2,511) showed a monotonous decrease; 12.5% (866) showed a local maximum; about 0.1% (6) showed a local minimum (Fig. 3; Supplementary File 1, Sheets 5–8).

The genes with a monotonous increase or decrease, with respect to HGF dose, were compared with a previous study^51^ on MDCK cysts tested at a higher HGF concentration (100 ng/ml). By comparing our dataset to the top 10 ost-differentially-expressed genes in that study, we thus identified a group of DEGs common in both studies (Table 2).

GO terms of the genes showing monotonous increase were over-represented and enriched in biological processes including cellular component assembly involved in morphogenesis, intracellular transport and catabolic process etc. (Supplementary File 3, Sheet 2). The genes showing monotonous decrease were enriched in biological processes including nuclear pore organization, tRNA metabolic process, DNA replication initiation, tRNA modification and ncRNA processing, etc. (Supplementary File 3, Sheet 3). This suggests that genes involved in growth and metabolic processes get upregulated with the addition of HGF and that certain genes involved in transcriptional and translational regulation get downregulated.

### Hill coefficients of genes with monotonously increasing HGF-response function

**Figure 4:**
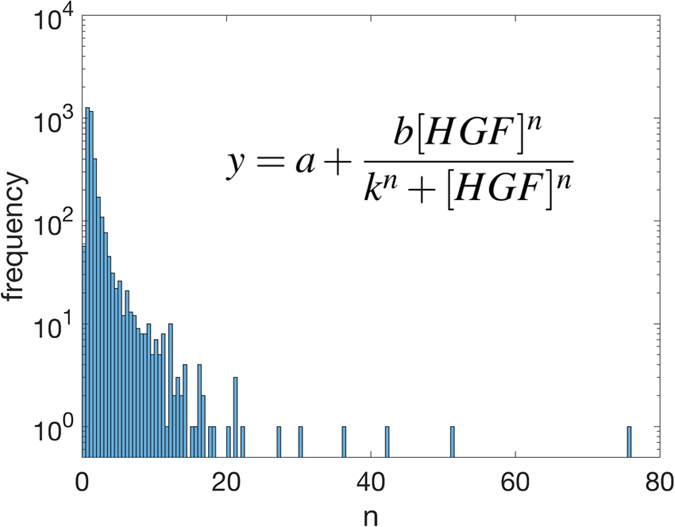
Distribution of Hill coefficients produced by MCMC fitting of the Hill equation to mapped, high-expressing, correlated, HGF-responsive genes. A parameter distribution is produced for each gene and we use 100,000 steps in our MCMC algorithm to ensure the accuracy of these parameter distributions. The mode is taken from these distributions and binned into bins of size 0.5. The inset shows the Hill equation used to fit the gene response, with the following parameters: *a*: basal expression, *b*: (maximum expression-basal expression), *k*: HGF value for half of maximum response, n: Hill coefficient.

The genes with monotonously increasing HGF dose-responses were selected for further study as these were most suitable for our downstream gene circuit engineering^28^. We therefore began a search for genes with ultrasensitive dose responses, defined as having a Hill coefficient (n) greater than one^1^. For each of the 3,527 monotonously increasing genes, we fitted a generic Hill function using a MCMC algorithm for curve fitting, as described in Methods. Overall, the resulting Hill coefficients showed a power-law distribution (Fig. 4), with many genes having relatively lower values of Hill coefficient and very few genes having very high Hill coefficients (maximum value of n = 76). There were 2,180 genes with n > 1 (Supplementary File 1, Sheet 9) and 1,347 with n ≤ 1 (which can correspond to negative cooperativity in the dose-response functions).

The dataset provided a number of interesting examples of ultrasensitive genes, including many previously-identified HGF-responsive genes (e.g. MMP1, TIMP1 and IL8^49^,^50^,^51^, etc.), as well as some unexpected ones. For example, the HGF receptor tyrosine kinase c-Met (MET_CANFA in Supplementary File 1, Sheet 9) is one of the high-expressing genes that increases monotonously with a 2-fold induction (154 to 291 FPKM, with a Hill coefficient of 1.09). This indicates a potential for positive feedback in HGF-c-Met mediated signalling, which has not been reported previously. We examined this further with a qRT-PCR experiment to see whether we could replicate the apparent ultrasensitive positive feedback (Fig. S5). The RNA-seq and qRT-PCR experiments agreed qualitatively: HGF does induce the HGF-receptor cMet and fitting the qRT-PCR data implied an even higher Hill coefficient of 3.46.

Our dataset also revealed that GO terms were over-represented and enriched for ribosomal RNA modifications (Supplementary File 3, Sheet 4), including genes involved in ribosomal RNA methyl transfer (e.g. TFB2M, FTSJ2, MRM1, DIMT1, METTL16, etc.). Overall, since the number of potentially ultrasensitive gene candidates was still very large, we adopted a further filtering process to select a subset for downstream verification.

### Validation of candidate genes with ultrasensitive responses to HGF

Out of the group of 2,180 genes with high Hill coefficients (n > 1), obtained by RNA-seq analysis, we verified selected candidate genes using quantitative real time PCR (qRT-PCR). We applied two selection criteria to narrow down the range of candidates: first, we selected genes with the highest fold-change between 0 ng/ml HGF and 16.7 ng/ml HGF. With a threshold log_2_ fold-change of 0.7, we retained 1,077 genes (Supplementary File 1, Sheet 10). Second, we selected for candidate genes with very low FPKM values (<50 in at least one of the replicates) in the 0 ng/ml HGF condition, resulting in 926 genes (Supplementary file 1, Sheet 11). These two criteria ensured that genes with high Hill coefficients had relatively low basal expression, with respect to overall response across conditions. Previous studies have shown that Hill coefficients obtained by fitting Hill functions to dose response curves with high basal expression, have low physiological relevance; in such cases, other methods of measuring sensitivity should be used^8^.

**Figure 5:**
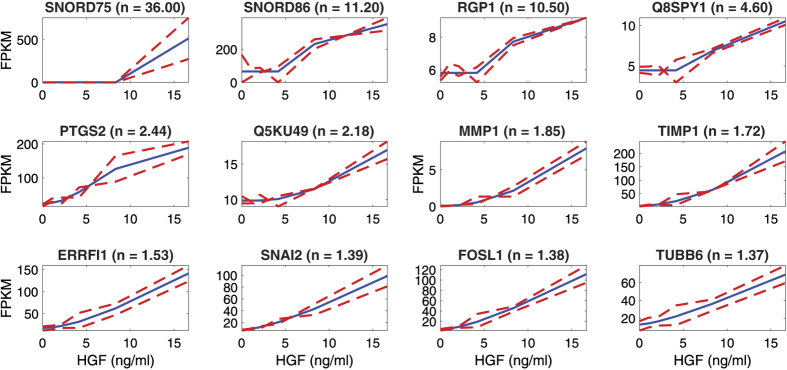
Fitting Hill functions to the RNAseq data and obtaining Hill coefficients for 12 HGF-responsive candidate genes. RNA-seq data of the biological replicates (two red dotted lines) and the corresponding Hill function fit (blue line). Hill functions were fitted by the MCMC algorithm (run for 100,000 steps) and the candidate genes are displayed in highest-to-lowest order of Hill coefficient (n). The titles show the gene name with the corresponding Hill coefficient in parenthesis.

Since our subset of 926 candidate ultrasensitive genes was still too large to verify in its entirety, we manually selected 12 genes with responses that span the observed range of fold-change values and Hill coefficients (Supplementary file 1, Sheet 12). The chosen genes were SNORD75, SNORD86, RGP1, Q8SPY1, PTGS2 (Q8SPQ9), Q5KU49, MMP1, TIMP1 (Q6QLW9), ERRFI1, SNAI2, FOSL1, and TUBB6 and had Hill coefficients spanning from n = 1.37–36.0 and FPKM values ranging from 0 to 756. The MCMC fittings of Hill functions to the FPKM values of these 12 genes are shown in Fig. 5 and the distribution profiles of the Hill coefficients for the 100,000 iterations of the MCMC fit is given in Fig. S6.

Within this dataset, we include the MMP1 promoter that we characterised previously^43^. MMP1 is a matrix metallopeptidase 1 protein involved in proteolysis of ECM and thus in cancer metastasis; HGF is known to induce cell scattering^64^. MMP1 has a particularly high fold-change upon HGF induction (10.2 log_2_ fold change). In fact, MMP1 is one of only 54 genes that goes up from <1 FPKM value at 0 HGF concentration to a high fold-change greater than 1.7 (Supplementary File 1, Sheet 13). TIMP1 (Q6QLW9_CANFA) is another gene with an ultrasensitive response and has a high induction level (~244 FPKM at 16.7 HGF concentration), albeit with a relatively high absolute basal level (~2.7 FPKM at 0 HGF concentration). Another interesting example is the SNORD75 small nucleolar RNA (predicted using sequences from RFAM and miRBase). It has low basal expression and high maximum FPKM (756) but its reliance on RNA pol III promoters may limit downstream use.

**Figure 6:**
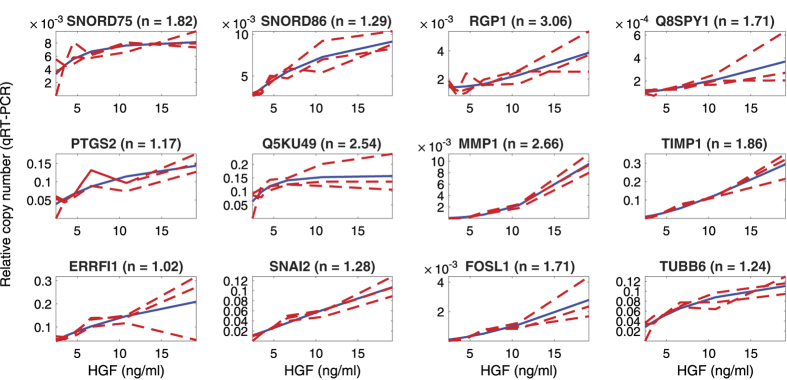
Validation of gene response found in RNAseq data using qRT PCR for 12 HGF-responsive candidate genes. qRT-PCR data for 3 biological replicates (red dotted lines) and the corresponding Hill function fit (blue line).

To verify the results, the MCMC fittings were repeated on independent data for the 12 genes, based on relative copy number data obtained by quantitative real time PCR (qRT-PCR), as shown in Fig. 6; the corresponding profiles of the Hill coefficients for the 100,000 iterations of the qRT-PCR MCMC fit is given in Fig. S7. Both the fits from RNA-seq data and qRT-PCR confirmed ultrasensitivity (n > 1) in all 12 genes. However, the two methods have different processing and sensitivity, so are only qualitatively comparable. There are thus discrepancies between the precise values of the apparent Hill coefficients. Moreover, HGF is a global regulator that is inherently noisy, so we expect a certain amount of batch-to-batch variation. To explore such variability further, we demonstrate the qualitative reproducibility with an additional set of qPCR data in biological triplicates (Fig. S8); the corresponding distribution profiles of the Hill coefficients for the 100,000 iterations of the MCMC fit are given in Fig. S9. These data represent independent experiments under conditions identical to those in main Fig. 6 and the two datasets are in qualitative agreement.

In comparison to the RNA-seq dataset, the apparent Hill coefficients were generally higher in the qRT-PCR data (range n = 1.02–6.76). However, the outlier SNORD genes and RGP1 did not display very high n-values in Fig. 6 and Fig. S8, mainly because the low HGF doses gave higher gene expression in the qRT-PCR assays. Overall, although Figs 5, 6 provide a range of ultrasensitive HGF-inducible genes, two candidates stand out: MMP1 and TIMP1 have relatively little variability within and between datasets and are thus good candidates for downstream engineering of ultrasensitive dose-response functions.

### Chromosomal positions of HGF-induced genes

The objective of the current study was to find genes with ultrasensitive behaviour, so that their regulatory elements could be exploited to design downstream engineering constructs. We reasoned that to integrate any HGF-responsive element in the MDCK genome, the best integration sites would be in the most HGF-activatable regions of the chromosomes. Regions with few HGF-activatable genes might promote constitutive activation or repression of an integrated transgene, or ORF connected to a local promoter-enhancer, and might thus be intrinsically less HGF-inducible, because of the local chromatin status. It should be noted that potential HGF-responsive chromosomal locations do not necessarily imply any altered ease or difficulty in targeted genome engineering; these would need to be tested individually. Overall, we aimed to find out if the response patterns of HGF-induced gene expression had genome locus-specific distributions. We therefore checked if there was any correlation between the expression pattern cluster and the chromosomal location.

**Figure 7:**
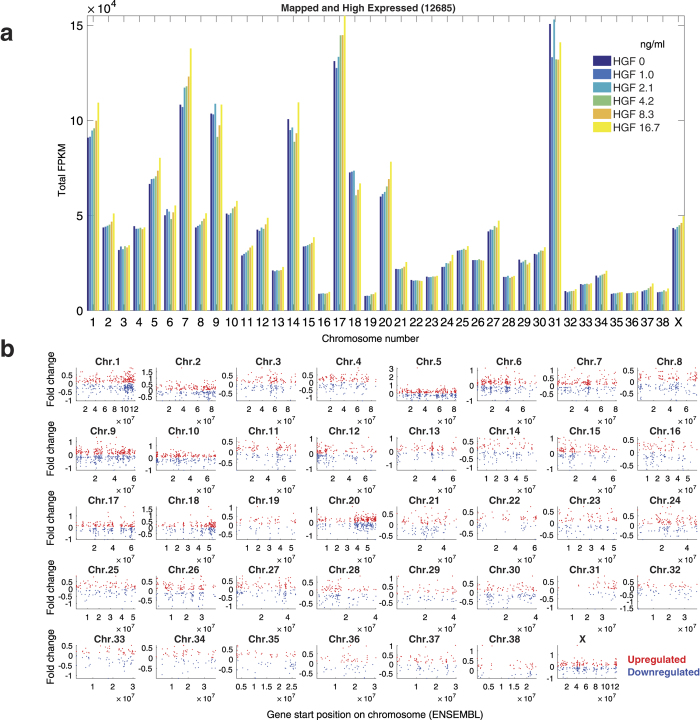
Distribution of gene expression across chromosomes. (**a**) FPKM values of all the mapped and high expressed genes (12,685) added together for an individual chromosome. The bar colors denote different HGF conditions as shown in the legend. (**b**) Log_2_ fold changes between 16.7 ng/ml and 0 ng/ml HGF exposure for all the responsive genes (6910) are indicated by red dots (upregulated) and blue dots (downregulated).

First, we found that the highly-expressed genes (12,685) were spread across all the chromosomes (Fig. 7a). The overall mapping showed little correlation to the size of the chromosomes and the number of genes per chromosome (Fig. S10). Chromosomes 1, 5, 7, 9, 14, 17, 18, 20 and 10 showed the highest mapping of reads and hence very high overall FPKM values, whereas chromosomes 13, 16, 19, 22–24, 26, 28, 29, 31–36 and 38 showed very low overall FPKM (<1 × 10^4^). Interestingly, a few chromosomes (e.g. 1, 5, 7, 17 and 20) appeared to increase in total FPKM with increasing HGF, indicating that these might be good choices for integrating constructs with HGF-dependent dose responses.

We next looked at the distribution of responsive genes (6,910) within chromosomes (Fig. 7b). The up- and down-regulated genes (red and blue dots, respectively) were fairly evenly distributed, although some chromosomes had dense patches of HGF-responsive genes (e.g. 1 and 20). It should be noted that there is no simple correlation between total FPKM levels per chromosome (Fig. 7a) and the number of differentially expressed genes (DEGs) (Fig. 7b). Rather, the DEGs are enriched in a chromosomes with quite different overall transcriptional activity. For example, even though the total expression level is higher for all six conditions in chromosome 31, there are fewer genes with differential expression when compared to chromosome 16. Furthermore, the distribution of ultrasensitive genes was spread across chromosomes, with some chromosomes (e.g. 1, 5, 20) displaying many ultrasensitive genes (Fig. S11). In summary, there are numerous genomic sites on these chromosomes that are potential target sites for integrating constructs with HGF-dependent dose responses, although ultrasensitive loci within chromosomes 1, 5 and 20 may be the best choices.

## Discussion

In this study, we profiled the transcriptomes of MDCK cysts grown in 3D culture, to identify HGF-responsive genes with varying degrees of ultrasensitivity. Overall, we identified ~12,000 genes (out of 25,000 annotated genes) that were differentially-expressed between the presence or absence of HGF. These included genes that were previously reported to be activated by HGF, including MMP1, TIMP1 and IL8^49^,^50^,^51^. By collecting gene expression data for various doses of HGF we were able to go one step further and characterise more detailed dose-response functions.

We found that HGF response functions could be placed into one of four qualitative classes, such as monotonous increase (activation; 3,527 genes), monotonous decrease (repression; 2,511 genes), local maximum (stripe; 867 genes) and local minimum (antistripe; 6 genes). As we were interested in ultrasensitivity, we focused on the largest gene class, monotonous increase in response to HGF, and we measured the nonlinearity of individual gene responses by fitting Hill functions and calculating the apparent Hill coefficients. The resulting Hill coefficients were distributed exponentially, with fewer genes having higher coefficients. We verified the dose-responses of these gene candidates using quantitative real time PCR (qRT-PCR) and found that, although the candidate genes showed similar dose response behaviours, the Hill coefficients varied somewhat for the two methods. Nonetheless, we were able to find 12 candidate genes that were consistently ultrasensitive (e.g. MMP1, TIMP1, SNORD75, etc; (Supplementary file 1, Sheet 12).

Interestingly, the HGF receptor tyrosine kinase c-Met is itself one of the high-expressing non-linear HGF-inducible genes. Consequently, there is a positive feedback in HGF-mediated c-Met signalling, that has not been previously reported. Conversely, there are reports showing downregulation of membrane-bound c-Met with increasing HGF, due to internalisation of HGF-bound c-Met receptor, resulting in ubiquitination and degradation^65^,^66^,^67^. This would suggest that the transcriptional upregulation of c-Met by HGF could be a mechanism of delayed compensation to maintain the c-Met receptor level at cell surface, without compromising on the dynamics of HGF signalling. These dynamics would be supported by the delays in transcription, translation and localisation of newly-induced c-Met, slowing its availability at the cell surface. As we can see, this study can give new insights regarding the responses of cells to HGF and this is important given its central role in metastasis, angiogenesis, tumorogenesis, mitogenesis and tissue regeneration^68^,^69^,^70^,^71^,^72^,^73^. The identification of dose-response aligned circuits in the HGF-cMET signalling pathway involved has important clinical implications^11^,^74^,^75^. Ultimately, as well as giving insights into HGF signalling, the dataset present here is a rich resource for engineering synthetic gene networks.

For synthetic biology applications, there are at least two ways to use the regulatory elements of the identified HGF-responsive genes. First, it is possible to isolate 1–2 kbp promoter region fragments, upstream of the transcription start site, because such fragments often retain suitably-responsive promoter-enhancer elements. For example, this was successfully achieved with a promoter fragment for MMP-1^43^. But it is worth noting that this methodology undermines the importance of genome locus-specific effects on ultrasensitive behavior of the HGF responsive genes. Alternatively, it is now possible to target genomes directly with site-directed nucleases, such as CRISPR-Cas9^76^. Using such technology, it is possible to integrate synthetic open reading frames just downstream of the transcription start site; this allows one to capture endogenous gene regulatory functions in their full promoter-enhancer contexts^77^. One limitation for this technology is the availability of PAM sites and the accessibility of Cas9 nuclease to chromatin^78^,^79^,^80^. Moreover, exploiting the regulatory function could still be a challenge in certain genes, where the ultrasensitive behavior is influenced by its ORF. For example, it is possible that for certain genes ultrasensitivity is related to control of RNA stability, rather than transcription. In such cases, a transgene would not necessarily inherit the ultrasensitive behaviour of the locus. Alternatively, the ultrasensitive response may be linked to a higher-dimensional location of the gene, or gene product, with similar issues for engineering transgene ultrasensitivity. Nonetheless, the data that we obtained in this study can therefore be used to inform future genome engineering based on endogenous HGF-responsive functions. Specifically, the four dose-response phenotypes (Fig. 3b), the distributions of their gene expression levels, and the relative activity of their corresponding chromosomal loci, are all valuable information for obtaining finely-tuned dose-response functions in response to HGF.

Tuning the response of a network or system is essential in synthetic biology and there are many challenges specific to engineering within living organisms. First, biological systems are very different from electronic or mechanical systems as they are noisy and changing constantly under evolutionary pressure. Furthermore, even though smaller modules like DNA parts (promoters, regulatory regions, coding regions) have been successfully identified and used interchangeably in different contexts, it is still a challenge to identify transferable functions that are free of their genetic and cellular contexts. To achieve this, one branch of synthetic biology aims to identify orthogonal components that are essentially independent from each other and the cell, i.e. they do not cross-react^81^,^82^,^83^,^84^,^85^. The advantage of this approach is that the parts can be treated as generic components, much like individual electronic logic gates on circuit boards. However, it is still unclear to what degree such systems will scale; it becomes progressively more difficult to identify increasing numbers of orthogonal components within a single cell, and they all add to metabolic load or burden^86^. A different way to work around this problem is to build one signaling network per cell and allow a combination of them to communicate with each other^87^, or identify suitable endogenous functions that already exist in a cell, embedded in the gene expression networks and metabolism, and exploit them within their natural contexts. Consequently, in this study, we identified a large number of genes that respond to extracellular HGF addition in a nonlinear manner, displaying varying response functions and degrees of ultrasensitivity. Ultimately, we believe that the dataset presented here will provide a valuable resource for those wishing to study and re-engineer HGF signalling.

## Methods

### Cell culture

MDCK type II cells were cultured in MEM with 10% FBS, 100 IU/ml penicillin, 100 mg/ml streptomycin, and 2 mM L-Glutamine at 37 °C, 5% CO_2_. For 3D MDCK cysts, adherent cells grown in 2D were digested with trypsin and resuspended to 4 × 10^4^ cells/ml in a type I collagen solution containing PureCol (2 mg/ml), Advanced BioMatrix 5005-B, 10X MEM, NaHCO_3_ (2.35 mg/ml), L-Glutamine (29.2 mg/ml), and 1 M HEPES (pH 7.6). About 40,000 cells in suspension were added to each well, containing a cell free collagen mix (polymerized previously at 37 °C). These were incubated at 37 °C (without CO_2_) for an hour. 1 ml of liquid media was added above the collagen layers and replaced every 2–3 days.

### HGF treatment and RNA isolation

Seven days after seeding, six different concentrations of HGF (2-fold dilution series) were added to the MDCK cysts resulting in final concentrations of 0, 1.0, 2.1, 4.2, 8.3, and 16.7 ng/ml. Total RNA was isolated 12 hours after HGF induction. To isolate total RNA from 3D cysts, the top collagen layer was peeled off and 350 μl of RLT buffer was added directly to the exposed cysts. The mixture was resuspended gently and processed according to the manufacturer’s protocol (RNeasy Miniprep kit from Qiagen). Total RNA concentrations were measured using a NanoDrop 1000 spectrophotometer and the quality was assessed using Agilent Bioanalyser before sending it for RNA sequencing or performing quantitative real time PCR (qRT-PCR). All samples had RNA Integrity Number (RIN) above 9.0.

### RNA sequencing

Libraries were prepared using the Illumina TruSeq Stranded mRNA Sample Preparation Kit v2, according to the manufacturer’s protocol. 500 ng of total RNA were used for poly(A)-mRNA selection using streptavidin-coated magnetic beads and were subsequently fragmented to approximately 300 bp. cDNA was synthesized using reverse transcriptase (SuperScript II, ref. 18064–014, Invitrogen) and random primers. The second strand of the cDNA incorporated dUTP in place of dTTP. Double-stranded DNA was further used for library preparation. dsDNA was subjected to A-tailing and ligation of the barcoded TruSeq adapters. All purification steps were performed using Ampure XP beads. Library amplification was performed by PCR on the size-selected fragments using the primer cocktail supplied in the kit.

Final libraries were analyzed using Agilent DNA 1000 chips to estimate the quantity and check size distributions, and these were then quantified by qPCR using the KAPA Library Quantification Kit (ref. KK4835, KapaBiosystems), prior to amplification with Illumina’s cBot. Sequencing was done with the Illumina HiSeq 2000 by pooling 4 random samples at equimolar ratios, distributed across eight lanes of the flow cells. Thus, single read lengths of 50 bp with a six base index were obtained.

### Sequence data processing

The sequenced reads were checked for quality using FastQC (http://www.bioinformatics.babraham.ac.uk/projects/fastqc/) and then mapped to the Ensembl version of the canine genome CanFam3.1 from iGenomes^88^,^89^,^90^, using Bowtie v2.1.0.0 and TopHat v2.0.8b, with default parameters. Alignments were converted to SAM format using SAMTools v0.1.19.0. FPKM values were calculated from the mapped reads using Cufflinks v2.1.1. RNA-seq data (Raw and FPKM values) along with their FastQC files are available at the Annotare-ArrayExpress database (www.ebi.ac.uk/arrayexpress) under the accession number E-MTAB-4959.

### Differential gene expression and statistical analysis

The Kruskal-Wallis test is a nonparametric (distribution free) test, and is used when the assumptions of ANOVA are not met. Both tests assess for significant differences on a continuous dependent variable by a grouping independent variable (with three or more groups). In ANOVA, we assume that distribution within each group is normally distributed and that there is approximately equal variance on the scores for each group. By contrast, in the Kruskal-Wallis Test, does not require any of these assumptions and was therefore used for comparing the non-normally-distributed datasets for each HGF-dose.

### Gene ontology statistical over-representation test

Ensembl ids of genes belonging to particular filtered class (e.g., responsive, monotonously increasing or decreasing) were submitted in the PANTHER classification system^91^,^92^ interface of the GO consortium website (http://geneontology.org/). The results of the corresponding GO term overrepresentation were obtained with default parameter values for the reference list of biological processes in *Canis lupus familiaris*. Only GO terms with significant (*p*-value < 0.05) fold enrichments were considered (Supplementary File 3).

### Curve fitting

Hill function parameters were fitted using a Metropolis Hastings Markov chain Monte Carlo (MCMC) method implemented in Matlab (Mathworks). MCMC methods are a class of sampling algorithms used to obtain a sequence of random samples from a probability distribution. They are generally used when direct sampling from the probability distribution is difficult. MCMC methods have advantages over other parameter estimation algorithms, such as Gradient descent, because they avoid the problem of getting trapped in local optima. There are several different MCMC methods, but in general they work by constructing a Markov chain whose equilibrium distribution equals the target probability distribution. As the number of steps increases, the sample more closely matches the target distribution. We used 100,000 steps to ensure the accuracy of our parameter distributions.

### Quantitative Real Time PCR (qRT-PCR)

Total RNA samples, collected from MDCK cysts, were first treated with DNaseI. Next, first strand synthesis was carried out using reverse transcriptase (SuperScript III, First Strand Synthesis mix, Invitrogen), according to the manufacturer’s protocol. Reverse transcribed cDNA was treated with RNase to remove all traces of RNA. Equal amounts of cDNA were used for all six conditions, when setting up real time PCR reactions. The RNase-treated cDNA was mixed with 2X concentrated Roche Light Cycler 480 SYBR Green I Master Mix and gene specific primers, according to the manufacturer’s protocol. qRT-PCR was performed using a Light Cycler 480 with standard cycling conditions. The relative copy numbers (2^−∆Ct^) were calculated from the C_t_ values obtained using the LC 480 program and were normalized to the Ubiquitin housekeeping gene. All real time PCR studies were performed with three biological and three technical replicates. Primer sequences for the tested genes and their respective amplicon lengths are available in (Supplementary file 4).

## Additional Information

**Acession codes**: ArrayExpress database (http://www.ebi.ac.uk/arrayexpress), accession codes E-MTAB-4959.

**How to cite this article**: Senthivel, V. R. *et al*. Identifying ultrasensitive HGF dose-response functions in a 3D mammalian system for synthetic morphogenesis. *Sci. Rep.* **6**, 39178; doi: 10.1038/srep39178 (2016).

**Publisher's note:** Springer Nature remains neutral with regard to jurisdictional claims in published maps and institutional affiliations.

## Supplementary Material

Supplementary Information

Supplementary File 1

Supplementary File 2

Supplementary File 3

Supplementary File 4

Supplementary File 5

## Figures and Tables

**Table 1:** Gene ontology set enrichment of responsive genes (12,201) for biological processes.

Process	number of genes	Fraction of genes	Fraction of process
metabolic process (GO:0008152)	1678	49.80%	33.00%
cellular process (GO:0009987)	1175	34.90%	23.10%
biological regulation (GO:0065007)	515	15.30%	10.10%
localization (GO:0051179)	409	12.10%	8.00%
cellular component organization or biogenesis (GO:0071840)	310	9.20%	6.10%
developmental process (GO:0032502)	295	8.80%	5.80%
response to stimulus (GO:0050896)	234	6.90%	4.60%
multicellular organismal process (GO:0032501)	163	4.80%	3.20%
immune system process (GO:0002376)	129	3.80%	2.50%
apoptotic process (GO:0006915)	70	2.10%	1.40%
biological adhesion (GO:0022610)	56	1.70%	1.10%
reproduction (GO:0000003)	35	1.00%	0.70%
locomotion (GO:0040011)	10	0.30%	0.20%
growth (GO:0040007)	1	0.00%	0.00%
cell killing (GO:0001906)	1	0.00%	0.00%

**Table 2:** Differentially expressed genes for HGF induction found in our study compared to *Chacon-Heszele, M.F. et al.*

Gene name (Upregulated)	Fold change (Our Study)	Fold change (Chacon-Heszele, M.F. *et al*.)	Gene name (Downregulated)	Fold change (Our Study)	Fold change (Chacon-Heszele, M.F. *et al*.)
'MMP1'	10.1951384	74.6253	**'CCL7'**	−2.120149856	−65.0118
'MAP3K5'	1.396876828	24.5208	**'CCL2'**	−3.914476279	−53.8085
'TXNRD1'	1.13790839	19.466	**'KLHL24'**	−2.44713517	−48.1247
'WDR37'	1.048303964	18.4215	**'FILIP1L'**	−2.021074158	−33.5399
'LRRC59'	2.076021878	16.8271	**'DAO'**	−1.789334105	−31.333
'FANCM'	1.938547434	11.6979	**'AGPHD1'**	−1.578062196	−24.6451
'FARSA'	1.585387481	10.3604	**'TMEM234'**	−1.508763067	−19.818
'SACS'	2.766298677	8.952	**'LRRC19'**	−2.326387874	−19.0976
'SLC25A32'	1.550329025	8.36223	**'HBP1'**	−2.054293316	−18.8617
'STK4'	1.474288553	7.68948	**'PARP9'**	−1.803372131	−18.6741
'PDK4'	2.025472157	7.5243	**'STAP2'**	−0.73106099	−17.7161
'TOMM40'	2.259897507	7.22431	**'ENOSF1'**	−1.578741283	−15.7093
'MPP6'	2.416929671	7.00514	**'HLA-DMA'**	−1.564983338	−14.9005
'DDX27'	1.354111753	6.72183	**'TMEM37'**	−1.554514417	−13.5176
'GNL3'	1.356639128	6.69788	**'VLDLR'**	−1.39548858	−13.5103
'PTER'	1.200079074	6.43559	**'PCMTD2'**	−1.036386665	−12.974
'ECE2'	2.026954143	6.12386	**'CCNG2'**	−1.767707003	−12.3896
'DOHH'	1.500344387	6.00274	**'ANKRD1'**	−0.598202926	−12.3206
'ZMYND19'	1.088635376	5.99707	**'DTX3L'**	−1.300645998	−11.2001
'FAM114A2'	0.879637749	5.94761	**'FYCO1'**	−1.564325433	−10.3473
'RBM28'	2.115158338	5.74236	**'PDCD4'**	−2.257106948	−9.54413
'SYNCRIP'	1.18594305	5.64882			
'VASH2'	0.965771621	5.64609			
'DNTTIP2'	0.773791986	5.62785			
'BLM'	2.316168912	5.4452			
'DDX18'	1.525705842	5.28791			
'PAM16'	1.034050333	5.24061			
'SDAD1'	0.857839191	5.2358			
'ITGB1'	0.689329879	5.17063			
'IMMT'	0.298192893	5.13259			
