# Supplementary material for: Identifying ultrasensitive HGF dose-response functions in a 3D mammalian system for synthetic morphogenesis
Source: Sci Rep. 2016 Dec 16;6:39178. doi: 10.1038/srep39178 (PMC5159920; doi:10.1038/srep39178)
Supplement: Supplementary Information [file srep39178-s1.pdf]

Supplementary Information

Identifying ultrasensitive HGF dose-response functions in a 3D mammalian system for synthetic morphogenesis

Vivek Raj Senthivel, Marc Sturrock, Gabriel Piedrafitta, Mark Isalan

Supplementary Figures and legends

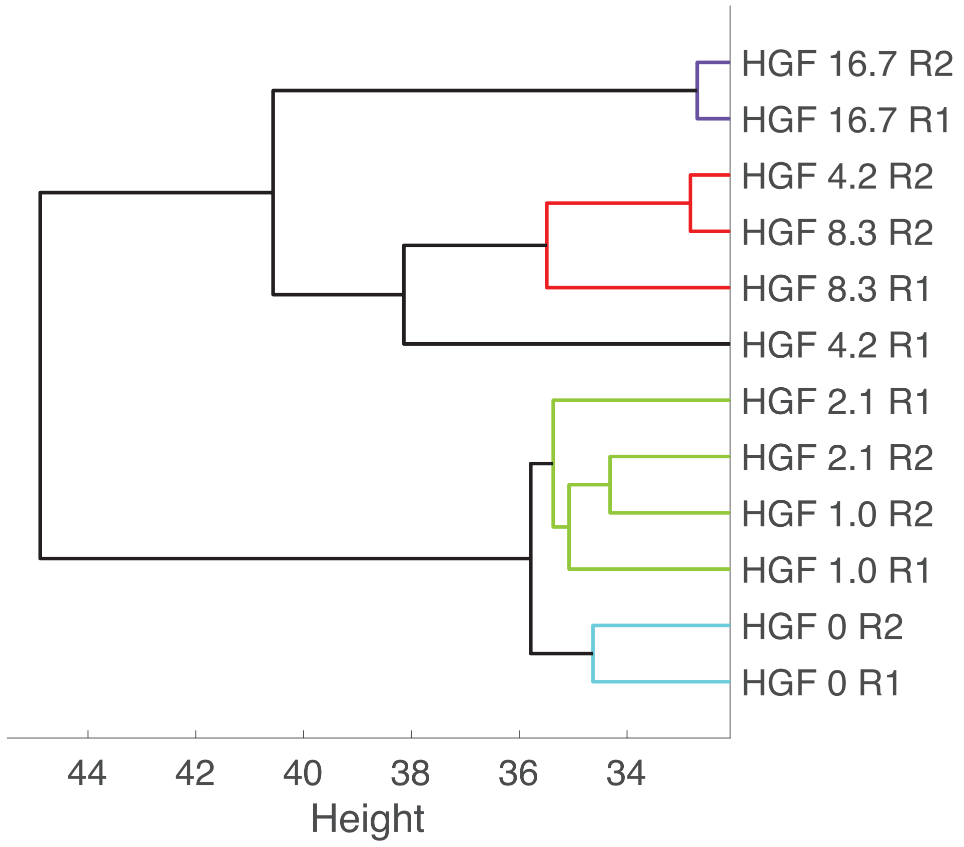

Figure S1. Hierarchical clustering of all RNA-seq data for both biological replicates using average linkage. The height denotes the degree of similarity between objects and the branches are color-coordinated so that branches whose linkage distance is less than a threshold of 35.5 are given the same color. Four distinct clusters were found corresponding to 0 ng/ml HGF (blue), 1.0 and 2.1 ng/ml HGF (green), 4.2 and 8.3 ng/ml HGF (red) and 16.7 ng/ml HGF concentrations (purple).

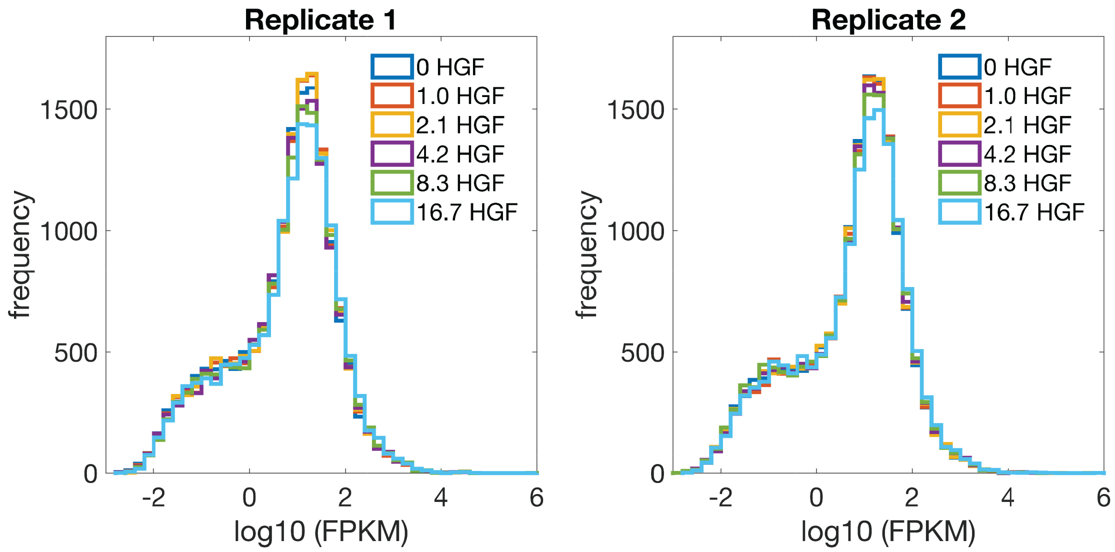

Figure S2. Histograms displaying log10 of the FPKM data for each biological replicate. The colors correspond to different experimental conditions which are displayed in the legends. The FPKM density distribution shows a bimodal distribution with a 'shoulder' of low-expressing genes followed by a peak of high-expressing genes.

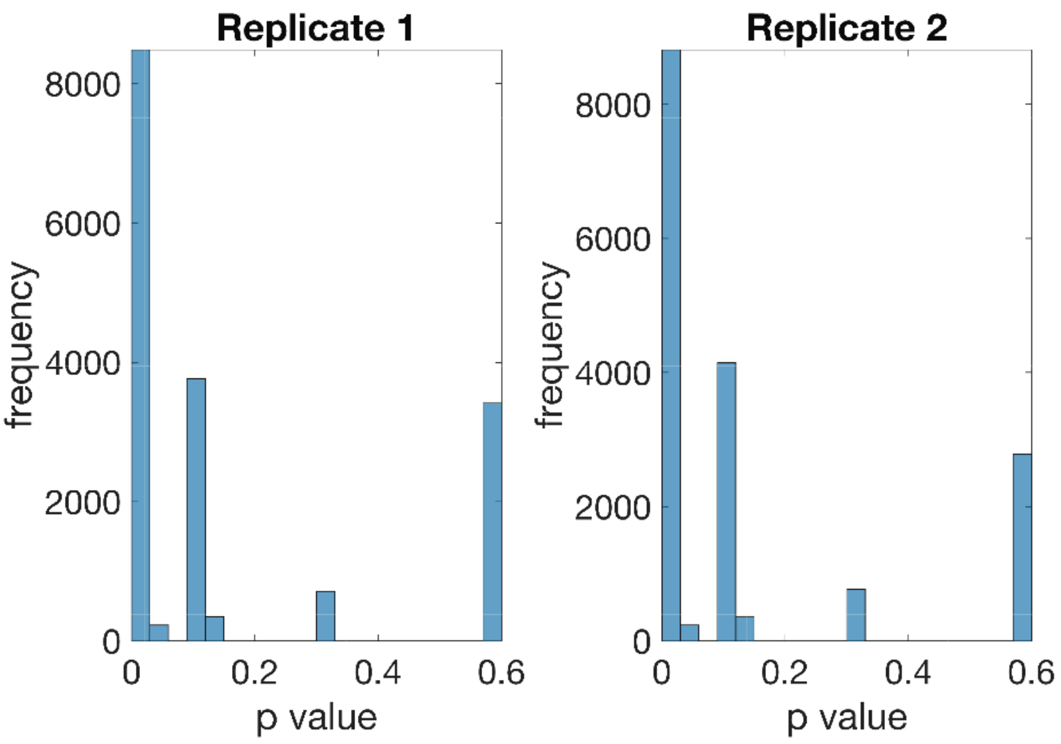

Figure S3. Histograms of *p*-values produced by a Kruskal Wallis test for each replicate. The Kruskal Wallis test compares data from mapped, high-expressing genes (12,685) for all six conditions, with a vector of the 0 ng/ml HGF condition repeated six times. If a gene does not respond to HGF stimulation then a high *p*-value is returned, indicating that there is no significant response. Genes with *p*-value below 0.33 were considered responsive (12,201).

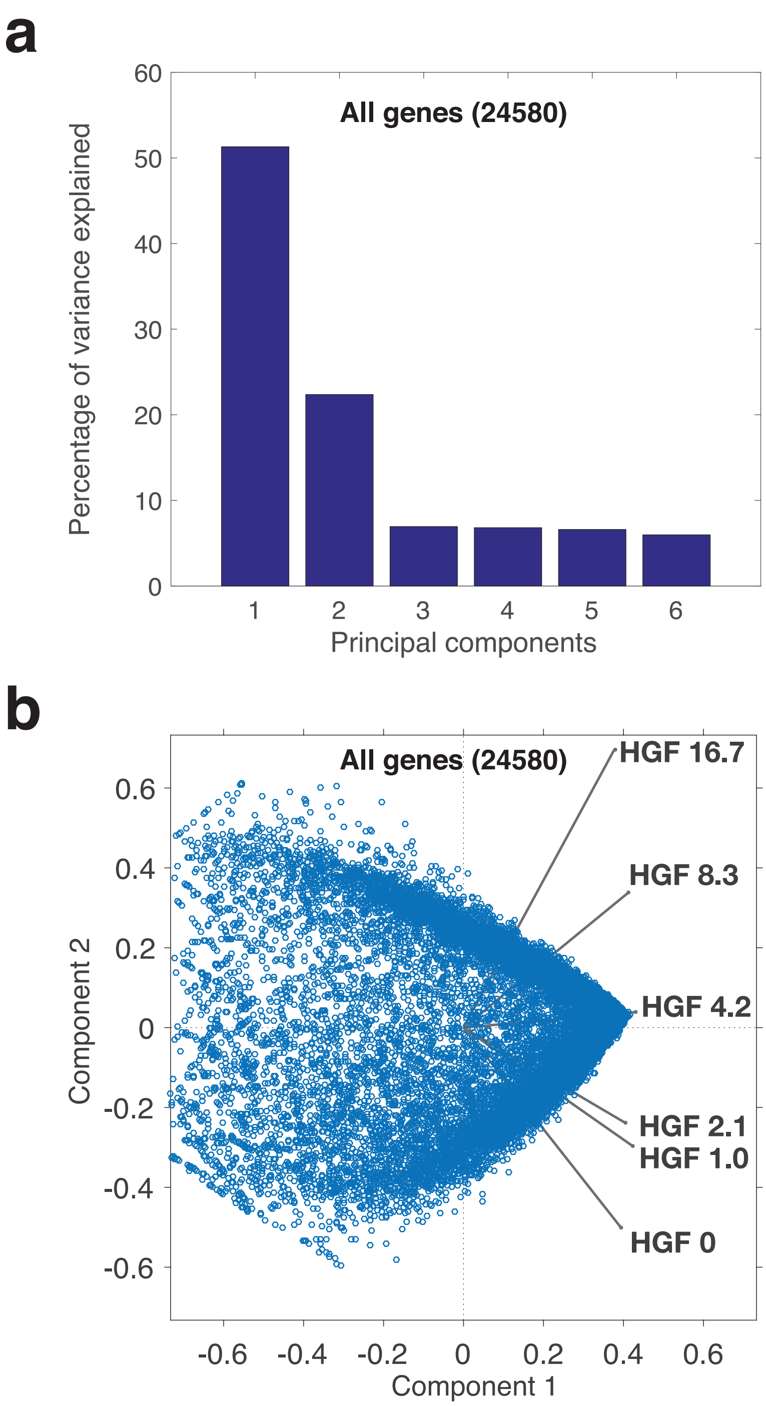

Figure S4. Principal component analysis of all genes (24,580). a. Percentage of variance explained by various components. The first component could explain 52% of variance in the data, whereas the second component could explain about 22% of variance in the data. **b.** Biplot showing component scores (red dots) of all the genes in a 2D plane of component 1 and component 2, and vector amplitudes (black lines) of the 6 conditions with respect to the first two principal components.

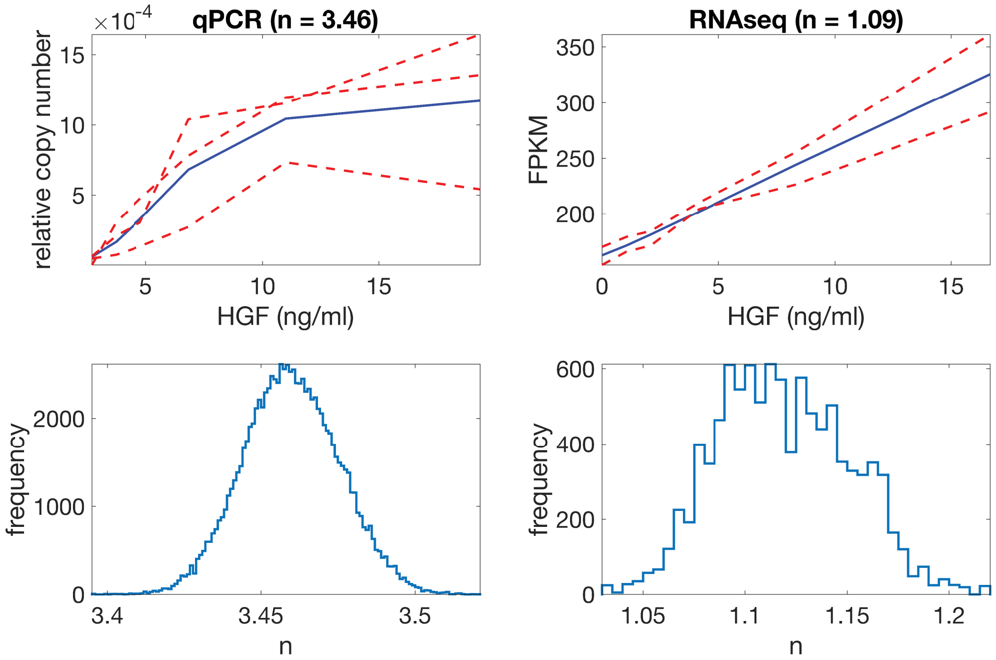

Figure S5. Dose response of HGF cell surface receptor cMet. cMet expression levels increase monotonously with increasing doses of HGF showing possible positive feedback by HGF signaling. The apparent Hill coefficients are 1.09 (RNAseq) and 3.46 (qPCR), as calculated by MCMC fitting of a Hill function (blue line) to biological replicates (red dotted lines).

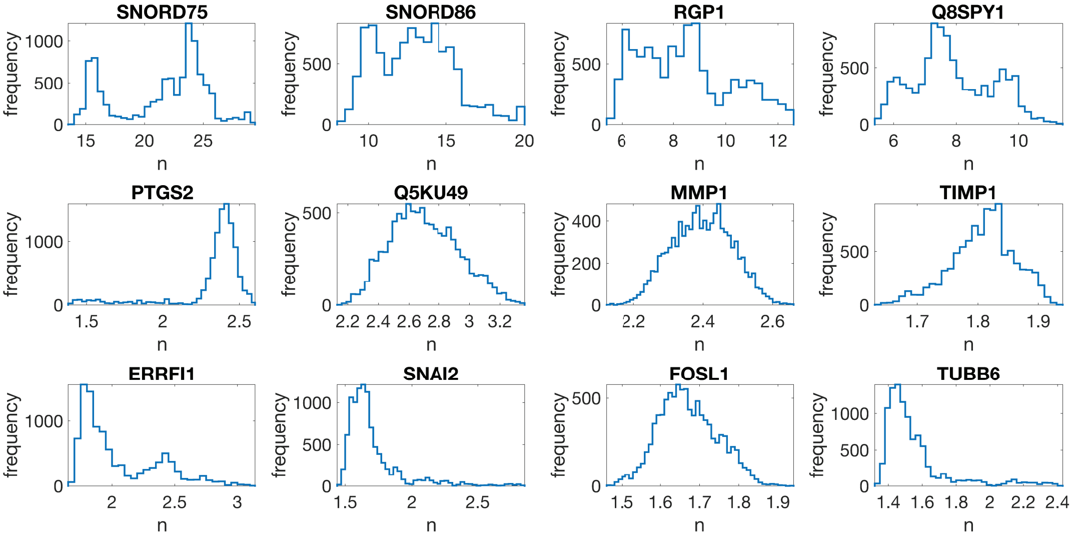

Figure S6. Hill coefficient distributions produced by fitting Hill functions to RNA-seq data. An MCMC algorithm was used (run for 100,000 steps), for 12 candidate genes, and results are displayed in highest-to-lowest order of Hill coefficient. The gene names are displayed in the titles. The mode of each distribution was used as the Hill coefficient value for each gene.

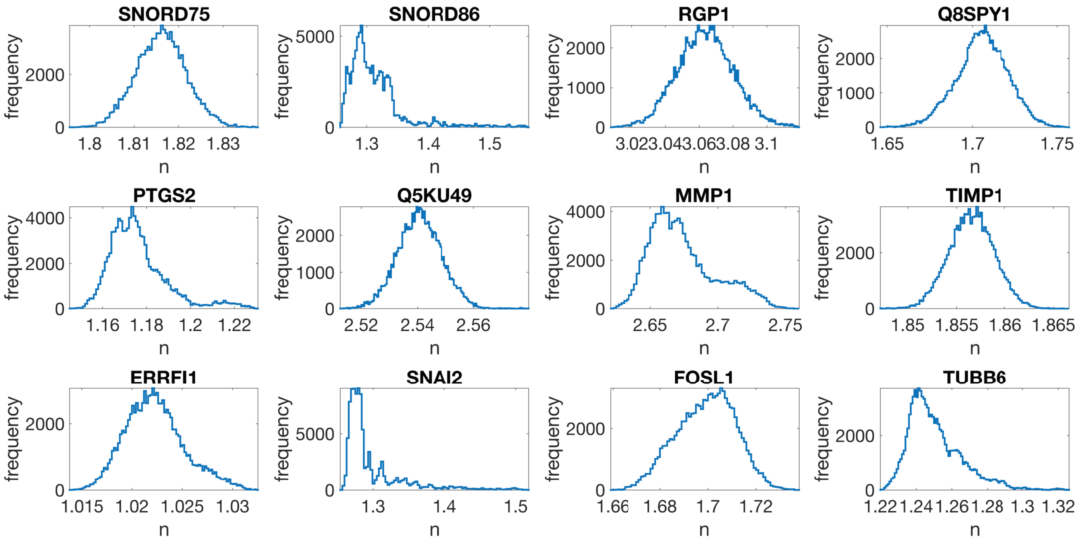

Figure S7. Hill coefficient distributions produced by fitting Hill functions to qRT-PCR data in Fig. 6. An MCMC algorithm was used (run for 100,000 steps), for 12 candidate genes, and results are displayed in order of highest-to-lowest Hill coefficient from previous RNA-seq data. The mode of each distribution was considered as the Hill coefficient value for each gene.

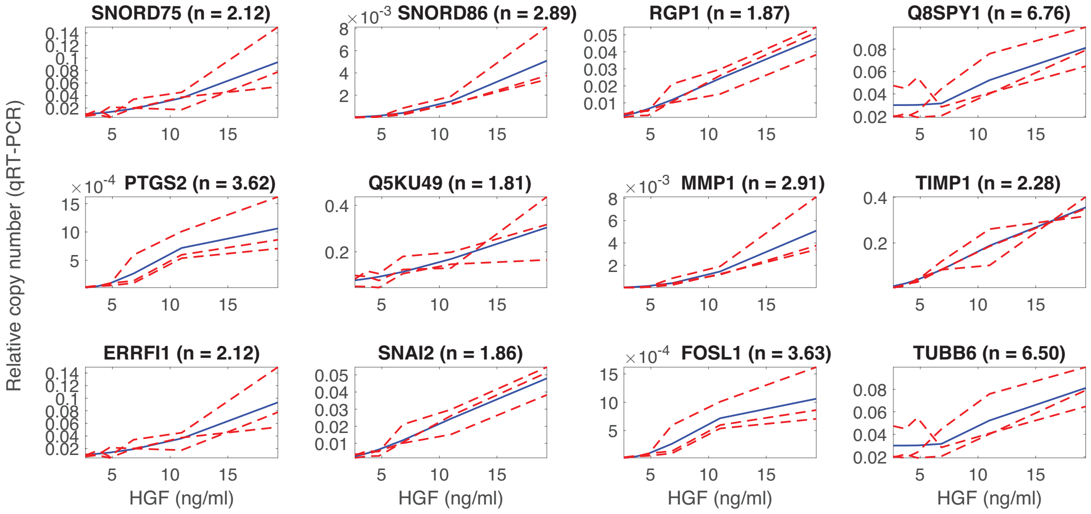

Figure S8. Validation of ultrasensitive gene responses found in RNAseq data using qRT PCR for 12 HGF-responsive candidate genes. qRT-PCR data for 3 biological replicates (red dotted lines) and the corresponding Hill function fit (blue line). The data represent independent experiments under conditions identical to those in main Fig. 6; the two datasets are in qualitative agreement.

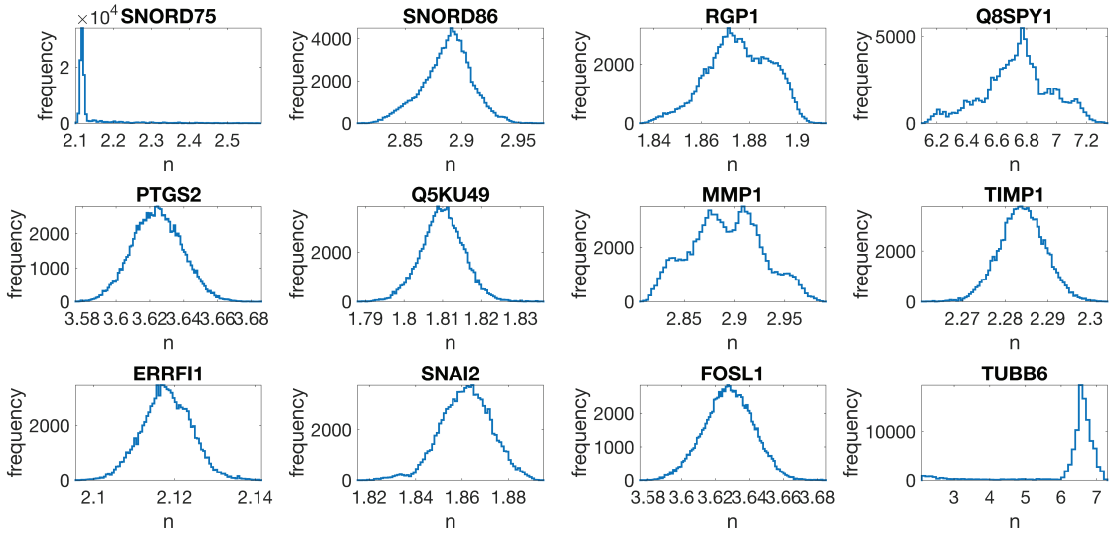

Figure S9. Hill coefficient distributions produced by fitting Hill functions to new qRT-PCR data in Fig. S8. An MCMC algorithm was used (run for 100,000 steps), for 12 candidate genes, and results are displayed in order of highest-to-lowest Hill coefficient from previous RNA-seq data. The mode of each distribution was considered as the Hill coefficient value for each gene.

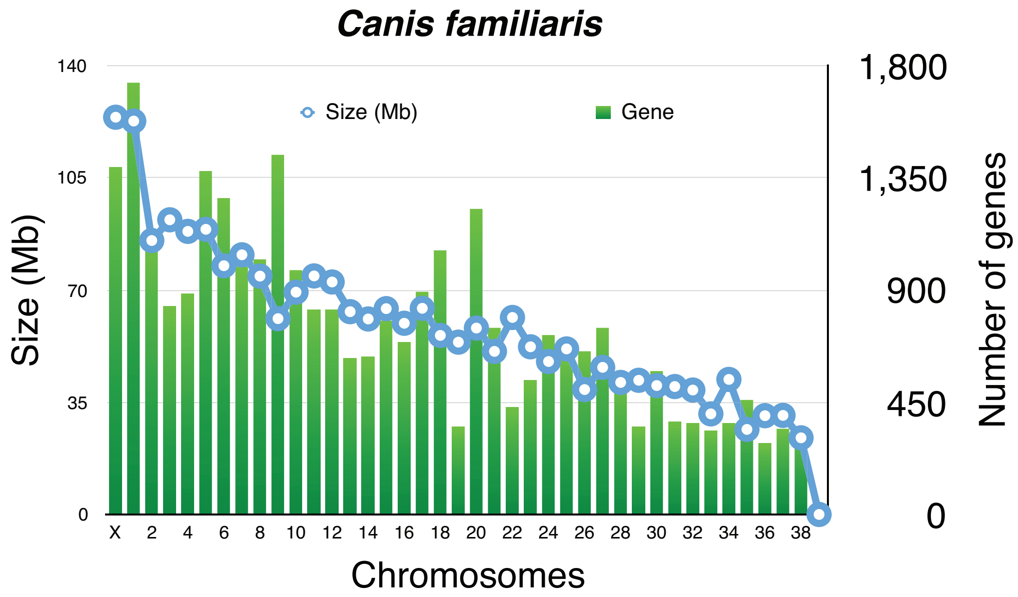

Figure S10. Summary of *Canine lupus familiaris* chromosomes:
The size of each chromosome in million bases (blue dots) and the number of genes (green bars) annotated on each chromosome (data obtained from the Dog Genome Sequencing Consortium: http://www.ncbi.nlm.nih.gov/genome/?term=txid9612[orgn]) [88-90]

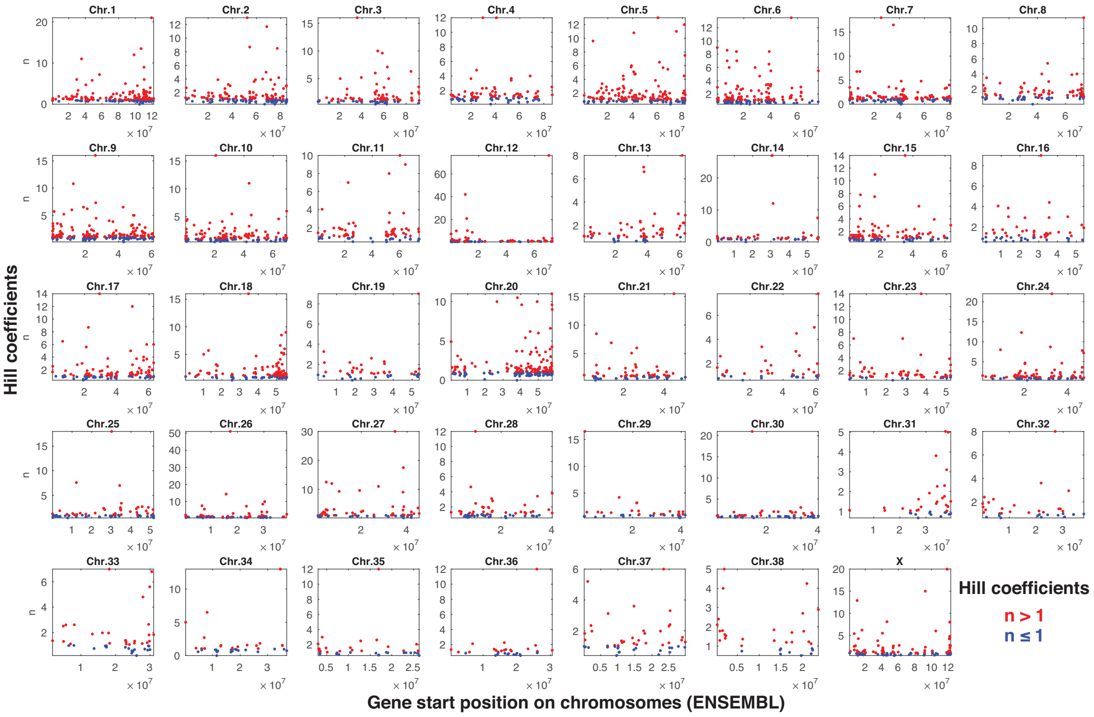

Figure S11. Distribution of Hill coefficients across chromosomes for the genes with a monotonous increase (3,527). The Hill coefficient of each gene is represented by red dots (n > 1) and blue dots (n ≤ 1).
